# Applying machine learning techniques to predict the risk of lung metastases from rectal cancer: a real-world retrospective study

**DOI:** 10.3389/fonc.2023.1183072

**Published:** 2023-05-24

**Authors:** Binxu Qiu, Zixiong Shen, Dongliang Yang, Quan Wang

**Affiliations:** ^1^ Department of Gastric and Colorectal Surgery, General Surgery Center, The First Hospital of Jilin University, Changchun, China; ^2^ Department of Thoracic Surgery, The First Hospital of Jilin University, Changchun, China

**Keywords:** machine learning, rectal cancer, lung metastasis, real world, web calculator

## Abstract

**Background:**

Metastasis in the lungs is common in patients with rectal cancer, and it can have severe consequences on their survival and quality of life. Therefore, it is essential to identify patients who may be at risk of developing lung metastasis from rectal cancer.

**Methods:**

In this study, we utilized eight machine-learning methods to create a model for predicting the risk of lung metastasis in patients with rectal cancer. Our cohort consisted of 27,180 rectal cancer patients selected from the Surveillance, Epidemiology and End Results (SEER) database between 2010 and 2017 for model development. Additionally, we validated our models using 1118 rectal cancer patients from a Chinese hospital to evaluate model performance and generalizability. We assessed our models’ performance using various metrics, including the area under the curve (AUC), the area under the precision-recall curve (AUPR), the Matthews Correlation Coefficient (MCC), decision curve analysis (DCA), and calibration curves. Finally, we applied the best model to develop a web-based calculator for predicting the risk of lung metastasis in patients with rectal cancer.

**Result:**

Our study employed tenfold cross-validation to assess the performance of eight machine-learning models for predicting the risk of lung metastasis in patients with rectal cancer. The AUC values ranged from 0.73 to 0.96 in the training set, with the extreme gradient boosting (XGB) model achieving the highest AUC value of 0.96. Moreover, the XGB model obtained the best AUPR and MCC in the training set, reaching 0.98 and 0.88, respectively. We found that the XGB model demonstrated the best predictive power, achieving an AUC of 0.87, an AUPR of 0.60, an accuracy of 0.92, and a sensitivity of 0.93 in the internal test set. Furthermore, the XGB model was evaluated in the external test set and achieved an AUC of 0.91, an AUPR of 0.63, an accuracy of 0.93, a sensitivity of 0.92, and a specificity of 0.93. The XGB model obtained the highest MCC in the internal test set and external validation set, with 0.61 and 0.68, respectively. Based on the DCA and calibration curve analysis, the XGB model had better clinical decision-making ability and predictive power than the other seven models. Lastly, we developed an online web calculator using the XGB model to assist doctors in making informed decisions and to facilitate the model’s wider adoption (https://share.streamlit.io/woshiwz/rectal_cancer/main/lung.py).

**Conclusion:**

In this study, we developed an XGB model based on clinicopathological information to predict the risk of lung metastasis in patients with rectal cancer, which may help physicians make clinical decisions.

## Introduction

Colorectal cancer is a prevalent gastrointestinal tumor with increasing incidence rates worldwide, causing over 900,000 deaths among almost 2 million new cases reported by the World Health Organization (WHO) (1, 2). In East Asia, where the disease is highly prevalent, the lifetime risk of developing colorectal cancer is 2% (2). Rectal cancer is a significant proportion of colorectal cancers, and early detection is crucial for improving patient outcomes. In Japan, early screening has increased the 5-year survival rate of rectal cancer patients to over 60%. However, late-stage rectal cancer, particularly with distant metastases, remains challenging to treat, with a survival rate of less than 15% (3). Unfortunately, lung metastases are a common site of metastasis, occurring in over 70% of patients within 5 years of diagnosis (4, 5). Although immunotherapy has improved survival rates, the benefits are limited, and systemic therapy and radiotherapy have low objective remission rates (6, 7). Early identification of high-risk patients with lung metastases from rectal cancer can improve survival quality and reduce unnecessary medical resource waste.

The integration of artificial intelligence with medical disciplines is growing, with machine learning playing a crucial role in this collaboration (8). Machine learning involves learning from data to improve algorithms, and resulting models can make predictions or decisions. Compared to traditional statistical methods, such as logistic regression, machine learning algorithms can analyze data associations more multidimensionally, making them particularly useful for analyzing complex medical data (9). Improved computing power and storage capacity have enabled machine learning to identify significant connections within medical data, facilitating personalized treatment recommendations, efficient healthcare delivery, and cost reduction (10, 11). Many researchers have used machine learning techniques for cancer metastasis assessment and early prediction with some success. However, these studies have had the disadvantage of using only public database data to develop models or having small sample sizes (12–14). Additionally, machine learning models are often viewed as “black boxes” and challenging for clinicians to understand and trust, hindering their widespread use in medical decision-making (15, 16).

The objective of our study was to use machine learning models to combine common clinicopathological factors and predict the probability of lung metastases in patients with rectal cancer. To validate our findings, we utilized external data from a Chinese hospital. Subsequently, we developed a web-based calculator using the most effective machine learning model. This predictive tool can help physicians assess the risk of lung metastasis in patients with rectal cancer and devise personalized medical strategies while optimizing medical resource allocation.

## Materials and methods

### Patient cohort

#### Development cohort

The Surveillance, Epidemiology, and End Results (SEER) database is a publicly available cancer reporting system that provides essential data for investigating complex diseases (17). After obtaining a license and permission, we generated a rectal cancer cohort using rectal cancer patient data from the SEER database. In this study, we included patient data from 2010 to 2017 as information on patients’ liver, brain, lung, and bone metastases was not collected into the database until after 2010 (18). Additional information about SEER is available on its official website (http://seer.cancer.gov/about/). To be included in our study, patients in the SEER database met the following criteria: 1. Pathologically diagnosed with rectal cancer based on the International Classification of Diseases morphological tumor code (ICD-O-3/WHO 2008); 2. Diagnosed between 2010 and 2017; 3. Rectal cancer as the primary tumor; 4. Complete clinicopathological information, including age, gender, race, marital status, T-stage, N-stage, pathological grading, carcinoembryonic antigen (CEA) levels, nerve invasion, tumor size, tumor deposits, primary site, and diagnostic information.

#### External validation cohort

**Figure 1:**
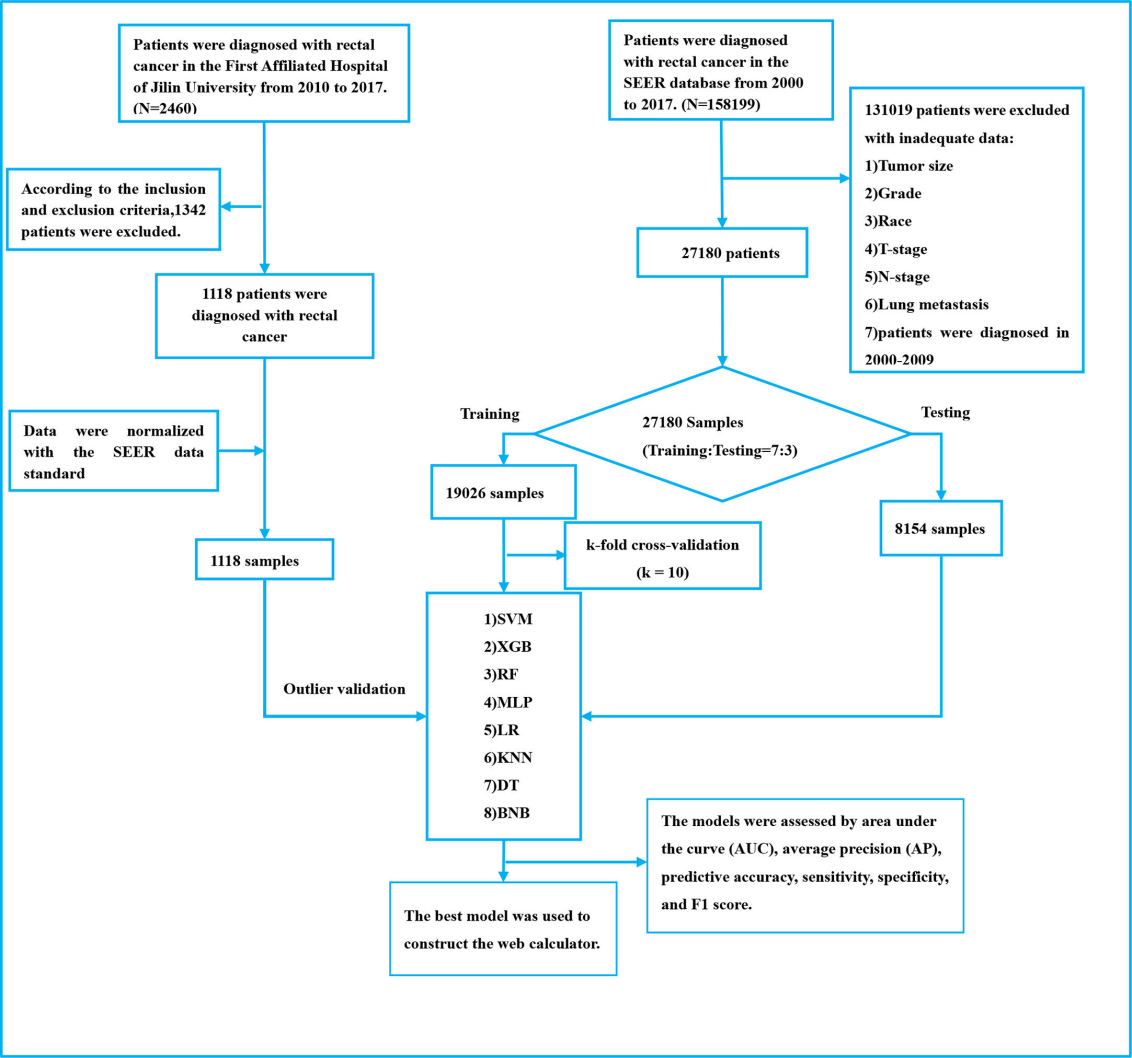
The Workflow diagram for study design and patient screening.

For external validation, we used data from 1,118 patients at the First Hospital of Jilin University, with additional criteria of no neoadjuvant radiotherapy before surgery and heterocoelous lung metastases (lung metastases occurring within 2 years of diagnosis of rectal cancer). The study was retrospective and did not involve patient safety or privacy, and an ethical exemption was granted. Please refer to **Figure 1** for a detailed outline of the patient selection process for both the development and external validation cohorts.

### Data collection and data processing

We utilized SEER * STAT (8.4.0) software to extract data from SEER Research Plus Data, 18 Registries + Hurricane Katrina Impacted Louisiana Cases + Hispanic Ethnicity, Nov 2020 Sub (2000-2018) for patients diagnosed with rectal cancer. The clinicopathological information from the external validation cohort was processed according to SEER standards (**Supplement Table 1**). The cases were staged using the 7th edition of the AJCC TNM staging and SEER-related guidelines. We categorized the variables for ease of use in model construction (**Supplement Table 2**).

### Construction of predictive models

We employed eight machine-learning algorithms for classification to predict the risk of lung metastasis in patients with rectal cancer. These algorithms included the extreme gradient boosting machine (XGB), random forest (RF), decision tree (DT), K-nearest neighbor (KNN), multilayer perceptron (MLP), logistic regression (LR), support vector machine (SVM), and Naive Bayes (BNB). XGB is a tree-based learning algorithm framework that has shown successful application in medical model construction in recent years (19). RF can reduce training variance, increase integration, and improve generalization by constructing multiple decision trees (20). DT is commonly used for high-accuracy tumor classification and image screening (21). KNN is a vital classification algorithm widely used for pattern recognition, data mining, and intrusion detection (22). MLP is a neural network model that can enhance pattern performance using stochastic gradient descent optimization with a momentum algorithm (23). LR is a classical binary variable classification algorithm commonly used in data mining due to its simplicity and greater explanatory power (24). BNB is a model based on an application of Bayes’ theorem that allows the use of continuous eigenvalues when they occur (25).

To train and validate our models, we randomly divided the rectal cancer patient data obtained from SEER into a training set and an internal validation set in a 7:3 ratio. Due to the significant impact of category imbalance on model performance when dealing with dichotomous variable problems, we addressed the data imbalance by using a synthetic minority category oversampling technique (SMOTE) to increase the number of patients with lung metastases from rectal cancer. This technique oversamples data samples from small categories to improve the model’s accuracy by increasing the number of data samples from small classes (26). We trained the eight models using the training set data. We used 80% and 20% of the data set for each set of parameters to fit the models for validation while searching for the optimal model parameters using random hyperparameters. We validated and evaluated the constructed models using the internal valid warranty data. Finally, we used an external validation cohort to determine the models’ extrapolation and generalization capabilities. We selected the best-performing model from the eight models mentioned above to construct the network predictor. The code for the article data analysis is represented in the supporting material (**Supporting Table 3**).

### Model performance and feature importance

In this study, we evaluated the performance of eight models using various metrics, including the area under the curve (AUC), the area under the precision-recall curve (AUPR), predictive accuracy, sensitivity, specificity, the Matthews correlation coefficient (MCC), and F1 score. The AUC value is typically calculated by Receiver Operating Characteristic (ROC) curve. Given the highly unbalanced nature of this dataset, we performed both PR curves, using the area under the PR curve as an essential metric for assessing model performance (27, 28). MCC is a particular case of the phi coefficient (φ). The True Class and Predicted Class are considered binary variables, and their correlation coefficients are calculated (similar to the correlation coefficient calculation between any two variables). The higher the correlation between the True and Predicted values, the better the prediction. A prediction will only yield a high score if it obtains good results in all four confusion matrix classes (29, 30). We used the Brier score to assess the accuracy of probabilistic predictions, which is suitable for tasks where the forecast must assign probabilities to mutually exclusive discrete outcomes. A lower score indicates a more accurate model (31). To evaluate the clinical value of the models, we used clinical decision curve analysis (DCA) (32). A calibration curve is essential for evaluating prediction models and assessing the difference and bias between the predicted values and actual observations (33). To analyze the importance of the included features across all algorithms, we used the permutation importance principle for feature importance analysis (34). This principle involves training the model, interrupting the data in one of the columns, and using that dataset to make predictions while assessing the decrease in prediction accuracy to reflect the importance of that feature variable. This process is then repeated for the other feature variables (35). The following formula was used to calculate model performance in this study:


$$\text{Accuracy}=\frac{TP+TN}{TP+TN+FP+FN}$$

$$\text{Precision}=\frac{TP}{TP+FP}$$

$$\text{Sensitivity}=\frac{TP}{TP+FN}=\text{recall}$$

$$\text{F}1=\frac{2*Precision*recall}{Precision+recall}$$

$$\text{Brier score}=\frac{1}{N}\;\sum_{T=0}^{n}(ft-Ot)$$

$$\text{MCC}=\frac{TP*TN-FP*FN}{\surd \left(TP+FP\right)\left(TP+FN\right)\left(TN+FP\right)\left(TN+FN\right)}$$

### Statistical analysis

All data analyses in this study were conducted using Python (version 3.8, Python Software Foundation) and R software (version 4.1.0). Continuous variables were reported as median and standard deviation, and group comparisons were performed using the Wilcoxon rank sum test. Categorical variables were reported as frequencies and percentages, and differences between groups were compared using the χ2 or Fisher’s exact test. Univariate logistic regression analysis was conducted for all variables included in the study. Variables with a two-sided P value< 0.05 were considered significant factors for lung metastasis from rectal cancer. Multivariable logistic regression was then performed to test whether these significant factors were independent risk factors for lung metastasis in patients with rectal cancer. The machine learning model included variables with a multivariable logistic regression p value< 0.05 for further analysis.

## Result

### Demographic composition and clinical baseline information

A total of 27,180 cases of rectal cancer diagnosed between 2010 and 2017 were included in this study using the SEER database. Among them, 912 cases (3.36%) had lung metastases, while 26,268 cases (96.64%) did not. Demographic and clinicopathological characteristics of all patients are reported in **Table 1**. The subjects were randomly assigned to a training set (n = 19026) and an internal test set (n = 8154) at a 7:3 ratio. An external test set comprising 1,118 patients with rectal cancer first diagnosed at our institution from 2010 to 2017 was also included. Detailed information regarding the training and test sets can be found in **Table 2**.

**Table 1:** Clinical and pathological characteristics of study population.

Variables	All	NLM	LM	P Value
	N=27180	N=26268	N=912	
**Age (mean (SD))**	62.50 (13.43)	62.52 (13.42)	61.85 (13.47)	0.138
Gender, n (%)				
** Male**	16007 (58.9)	15477 (58.9)	530 (58.1)	0.651
** Female**	11173 (41.1)	10791 (41.1)	382 (41.9)	
Race, n (%)				
** White**	21566 (79.3)	20835 (79.3)	731 (80.2)	0.539
** Black**	2669 (9.8)	2575 (9.8)	94 (10.3)	
** Asian or Pacific Islander**	2698 (9.9)	2620 (10.0)	78 (8.6)	
** American Indian/Alaska Native**	247 (0.9)	238 (0.9)	9 (1.0)	
Marital, n (%)				
** Married (including common law)**	15265 (56.2)	14812 (56.4)	453 (49.7)	0.001
** Single (never married)**	4497 (16.5)	4319 (16.4)	178 (19.5)	
** Widowed**	2864 (10.5)	2765 (10.5)	99 (10.9)	
** Divorced**	2743 (10.1)	2623 (10.0)	120 (13.2)	
** Unknown**	1440 (5.3)	1394 (5.3)	46 (5.0)	
** Separated**	293 (1.1)	281 (1.1)	12 (1.3)	
** Unmarried or Domestic Partner**	78 (0.3)	74 (0.3)	4 (0.4)	
T stage, n (%)				
** T1**	6529(24.0)	6391(24.3)	138(15.1)	<0.001
** T2**	4551 (16.7)	4498 (17.1)	53 (5.8)	
** T3**	13660 (50.3)	13136 (50.0)	524 (57.5)	
** T4**	2440 (9.0)	2243 (8.5)	197 (21.6)	
N stage, n (%)				
** N0**	16077 (59.2)	15796 (60.1)	281 (30.8)	<0.001
** N1**	8231 (30.3)	7775 (29.6)	456 (50.0)	
** N2**	2872 (10.6)	2697 (10.3)	175 (19.2)	
Grade, n (%)				
** Grade I**	3899 (14.3)	3844 (14.6)	55 (6.0)	<0.001
** Grade II**	19620 (72.2)	18944 (72.1)	676 (74.1)	
** Grade III**	3192 (11.7)	3029 (11.5)	163 (17.9)	
** Grade IV**	469 (1.7)	451 (1.7)	18 (2.0)	
Tumor Deposits, n (%)				
** No**	17159 (63.1)	16895 (64.3)	264 (28.9)	<0.001
** Yes**	2342 (8.6)	2221 (8.5)	121 (13.3)	
** Unknown**	7679 (28.3)	7152 (27.2)	527 (57.8)	
Perineural Invasion, n (%)				
** No**	17839 (65.6)	17453 (66.4)	386 (42.3)	<0.001
** Yes**	2328 (8.6)	2205 (8.4)	123 (13.5)	
** Unknown**	7013 (25.8)	6610 (25.2)	403 (44.2)	
CEA, n (%)				
** Negative**	8828 (32.5)	8694 (33.1)	134 (14.7)	<0.001
** Borderline**	83 (0.3)	83 (0.3)	0 (0.0)	
** Positive**	6944 (25.5)	6407 (24.4)	537 (58.9)	
** Unknown**	11325 (41.7)	11084 (42.2)	241 (26.4)	
**Tumor Size (mean (SD))**	4.04 (3.50)	3.97 (3.40)	5.91 (5.37)	<0.001

CEA, Carcinoembryonic antigen; LM, Lung metastasis; NLM, no lung metastasis.

**Table 2:** Clinical and pathological characteristics of the training set, test set and validation set.

Variables	SEER database(N=27180)		Outer validation (N=1118)	P Value
	Training (N=19026)	Testing (N=8154)		
**Age (mean (SD))**	62.52 (13.39)	62.44 (13.52)	62.78(13.50)	0.493
Gender, n (%)				
** Male**	11204(58.9)	4803(58.9)	653(58.4)	0.747
** Female**	7822 (41.1)	3351 (41.1)	465 (41.6)	
Race, n (%)				
** White**	15079 (79.3)	6487 (79.6)	0	<0.001
** Black**	1893 (9.9)	776 (9.5)	0	
** Asian or Pacific Islander**	889 (9.9)	809 (9.9)	1118(100.0)	
** American Indian/Alaska Native**	165 (0.9)	82 (1.0)	0	
Marital, n (%)				
** Married (including common law)**	10665 (56.1)	4600 (56.4)	1116(99.8)	<0.001
** Single (never married)**	3177 (16.7)	1320 (16.2)	0	
** Widowed**	1998 (10.5)	866 (10.6)	0	
** Divorced**	1930 (10.1)	813 (10.0)	0	
** Unknown**	996 (5.2)	444 (5.4)	0	
** Separated**	208 (1.1)	85 (1.0)	0	
** Unmarried or Domestic Partner**	52 (0.3)	26 (0.3)	2(0.2)	
T stage, n (%)				
** T1**	4476 (23.5)	2053 (25.2)	271 (24.2)	0.272
** T2**	3192 (16.8)	1359 (16.7)	183 (16.4)	
** T3**	9675 (50.9	3985 (48.9)	584 (52.2)	
** T4**	1683(8.8)	757 (9.3)	80 (7.2)	
N stage, n (%)				
** N0**	11224 (59.0)	4853 (59.5)	630 (56.4)	0.517
** N1**	5799 (30.5)	2432 (29.8)	380 (34.0)	
** N2**	2003 (10.5)	869 (10.7)	108 (9.7)	
Grade, n (%)				
** Grade I**	2688 (14.1)	1211 (14.9)	162 (14.5)	0.361
** Grade II**	13718 (72.1)	5902 (72.4)	826 (73.9)	
** Grade III**	2287 (12.0)	905 (11.1)	110 (9.8)	
** Grade IV**	333 (1.8)	136 (1.7)	20 (1.8)	
Tumor Deposits, n (%)				
** No**	12066 (63.4)	5093 (62.5)	629 (56.3)	<0.001
** Yes**	1637 (8.6)	705 (8.6)	128 (11.4)	
** Unknown**	5323 (28.0)	2356 (28.9)	361 (32.3)	
Perineural Invasion, n (%)				
** No**	12567 (66.1)	5272 (64.7)	674 (60.3)	<0.001
** Yes**	1639 (8.6)	689 (8.4)	89 (8.0)	
** Unknown**	4820 (25.3)	2193 (26.9)	355 (31.8)	
CEA, n (%)				
** Negative**	6227 (32.7)	2601 (31.9)	422 (37.7)	<0.001
** Borderline**	56 (0.3)	27 (0.3)	38 (3.4)	
** Positive**	4875 (25.6)	2069 (25.4)	326 (29.2)	
** Unknown**	7868 (41.4)	3457 (42.4)	332 (29.7)	
**Tumor Size (mean (SD))**	4.05 (3.50)	4.00 (3.50)	3.93 (3.82)	0.324
Lung Met, n (%)				
** No**	18401(96.7)	7867(96.5)	932(83.3)	<0.001
** Yes**	625(3.3)	287(3.5)	186(16.6)	

CEA, Carcinoembryonic antigen; SEER, The Surveillance, Epidemiology, and End Results (SEER) database.

### Univariate analysis and multivariable logistic regression analysis

Univariate logistic regression analysis of the training dataset revealed that marital status, T and N stage, preoperative CEA levels, tumor deposition, perineural invasion, and tumor size were significantly associated with lung metastasis in patients with rectal cancer (P<0.05; **Table 3**). Variables with a P-value of less than 0.05 in the univariate analysis were included in multivariable logistic regression analysis to identify independent risk factors for lung metastasis in rectal cancer patients. Multivariable logistic regression analysis revealed that T and N stage, CEA, tumor deposition, perineural invasion, grade, and tumor size were independent risk factors for lung metastasis in rectal cancer (P< 0.05; **Table 3**).

**Table 3:** Univariate analysis and multivariable logistic regression analysis of variables.

Variables	Category	Univariate Analysis	Multivariable Analysis	
		P value	Odds Ratio (95% CI)	P value
**Age**		0.096		
**Sex**	Male	0.506		
	Female			
**Race**	White	0.162		
	Black			
	Asian or Pacific Islander			
	American Indian/Alaska Native			
**Marital status**	Married	0.010	Reference	Reference
	Single (never married)		1.06 (0.88-1.28)	0.530
	Widowed		1.12 (0.89-1.41)	0.349
	Divorced		1.21 (0.98-1.51)	0.080
	Unknown		1.10 (0.80-1.52)	0.550
	Separated		1.08 (0.58-2.01)	0.806
	Unmarried or Domestic Partner		1.54 (0.52-4.55)	0.431
**Grade**	Grade I	<0.001	Reference	Reference
	Grade II		2.45 (1.82-3.31)	<0.001
	Grade III		2.56 (1.83-3.58)	<0.001
	Grade IV		2.01 (1.13-3.57)	0.017
**T stage**	T1	<0.001	Reference	Reference
	T2		0.61 (0.44-0.85)	0.004
	T3		1.05 (0.84-1.32)	0.676
	T4		1.51 (1.17-1.96)	0.002
**N stage**	N0	<0.001	Reference	Reference
	N1		2.37 (2.00-2.81)	<0.001
	N2		2.72 (2.17-3.40)	<0.001
**CEA Pretreatment**	Negative	<0.001	Reference	Reference
	Borderline		1.74 (0.79-2.69)	0.940
	Positive		3.63 (2.98-4.42)	<0.001
	Unknown		1.30 (1.04-1.62)	0.022
**Perineural Invasion**	No	<0.001	Reference	Reference
	Yes		1.57 (1.25-1.98)	<0.001
	Unknown		1.47 (1.23-1.75)	<0.001
**Tumor Deposits**	No	<0.001	Reference	Reference
	Yes		1.59 (1.25-2.01)	<0.001
	Unknown		4.41 (3.67-5.31)	<0.001
**Tumor size**		<0.001	1.03 (1.02-1.04)	<0.001

CEA, Carcinoembryonic antigen.

### Model performance

**Figure 2:**
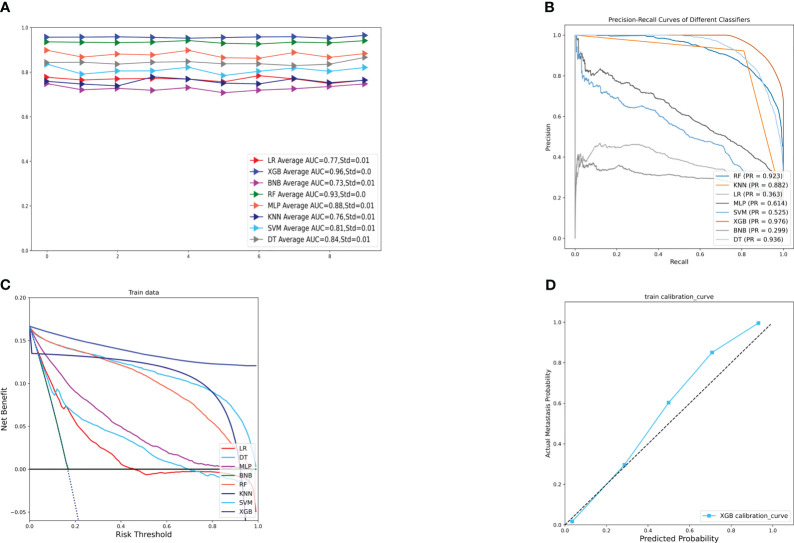
**(A)** Ten-fold cross-validation results of different machine models in the training set. **(B)** PR curves of different machine learning models in the training set. **(C)** DCA curves of different machine learning models in the training set. **(D)** Calibration curves of the best models in the training set. LR, logistic regression; DT, decision tree; RF, random forest; XGB, extreme gradient boosting;BNB, plain Bayesian classification; MLP, multilayer perceptron; SVM, support vector machine; KNN, k-nearest neighbor.

**Figure 3:**
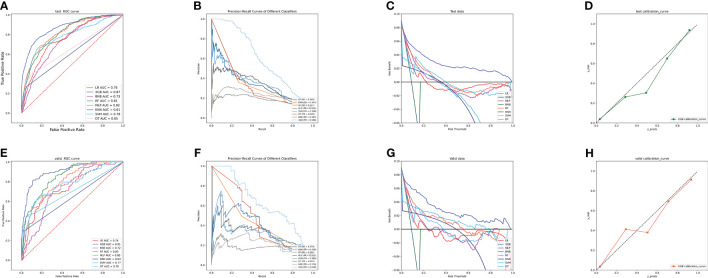
**(A)** ROC curves of different machine learning models in the internal validation set. **(B)** PR curves of different machine learning models in the internal test set. **(C)** DCA curves of different machine learning models in the internal test set. **(D)** Calibration curves of different machine learning models in the internal test set **(E)** ROC curves of different machine learning models in the external test set. **(F)** PR curves of different machine learning models in the external test set. **(G)** DCA curves of different machine learning models in the external test set. **(H)** Calibration curves of different machine learning models in the external validation set. LR, logistic regression; DT, decision tree; RF, random forest; XGB, extreme gradient boosting; NBC, plain Bayesian classification; MLP, multilayer perceptron; SVM, support vector machine; KMN, k-nearest neighbor; DCA, Decision curve analysis; PR, precision-recall.

**Figure 4:**
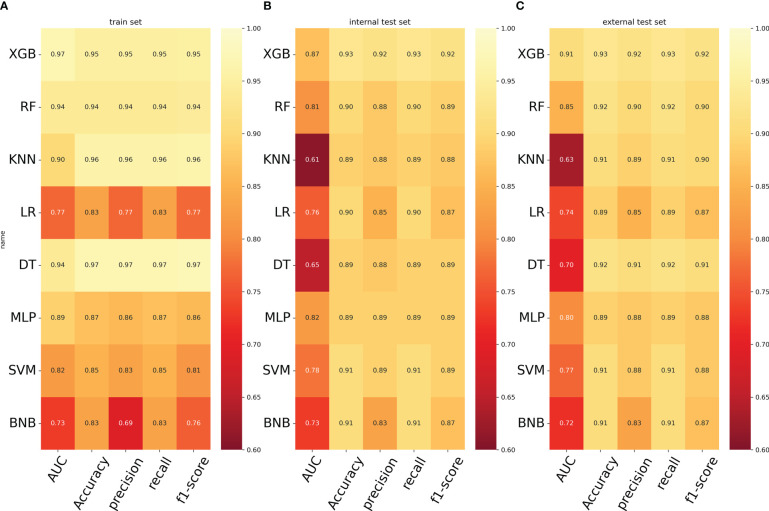
**(A)** Prediction performance of different models in the training set. **(B)** Prediction performance of different models in the internal validation set. **(C)** Prediction performance of different models in the external validation set. LR, logistic regression; DT, decision tree; RF, random forest; XGB, extreme gradient boosting; NBC, plain Bayesian classification; MLP, multilayer perceptron; SVM, support vector machine; KMN, k-nearest neighbor.

**Figure 2A** displays the results of the tenfold cross-validation, indicating that XGB exhibited the best performance with an average AUC value of 0.96 (std=0.00), surpassing other models such as RF (AUC=0.93, std=0.00), LR (AUC=0.77, std=0.01), SVM (AUC=0.81, std=0.01), MLP (AUC=0.88, std = 0.01), KNN (AUC = 0.76, std = 0.01), BNB (AUC = 0.73, std = 0.01), and DT (AUC = 0.84, std = 0.01). Moreover, the XGB model obtained the best AUPR and MCC in the training set, reaching 0.98 and 0.88, respectively (**Figure 2B**). The XGB model also demonstrated the lowest Brier score of 0.025 among all models. Based on the training set data, the DCA curves revealed that the XGB model had high reliability (**Figures 2C, D**). In the internal validation set, the XGB model achieved the highest AUC of 0.87 and exhibited high accuracy, precision, sensitivity, and F1 score (**Figures 3A**, **4**). In the external validation set, the XG B model attained the highest AUC of 0.91 and displayed excellent accuracy, precision, sensitivity, and F1 score (**Figures 3E**, **4**). Furthermore, the XGB model demonstrated a larger AUPR than other models (**Figures 3B, F**). The XGB model obtained the highest MCC in the internal test set and external validation set, with 0.61 and 0.68, respectively. The DCA and clinical decision curves show that the XGB model has good clinical decision-making ability and actual predictive power compared to the other seven models (**Figures 3C, G, D, H**). Considering the high predictive performance of the XGB model in both internal and external validation sets, we designate it as the best model.

### Relative feature importance on prediction

**Figure 5:**
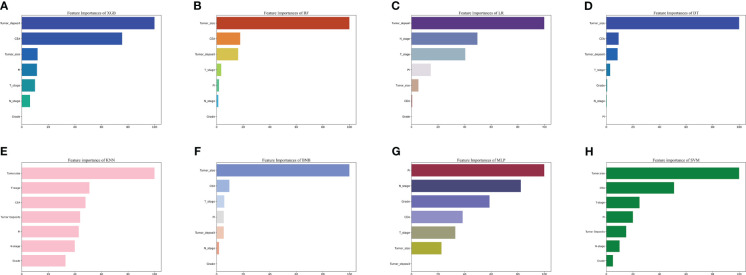
The importance of Variables in each prediction model. **(A)** Feature Importance of XGB. **(B)** Feature Importance of RF. **(C)** Feature Importance of LR. **(D)** Feature Importance of DT. **(E)** Feature Importance of KNN. **(F)** Feature Importance of BNB. **(G)** Feature Importance of MLP. **(H)** Feature Importance of SVM. LR, logistic regression; DT, decision tree; RF, random forest; XGB, extreme gradient boosting; BNB, plain Bayesian classification; MLP, multilayer perceptron; SVM, support vector machine; KMN, k-nearest neighbor; CEA, Carcinoembryonic antigen; PI, perineural invasion.

The importance of the features in predicting lung metastases was evaluated using the importance ranking principle, and the results are shown in **Figure 5**. Tumor size and deposits were found to be the most important variables across most machine-learning models. Conversely, differentiation grade was found to be the least important variable in most models, but it still contributed to the models to some extent. In the XGB model, the relative importance of features in descending order was tumor deposits, CEA, peripheral nerve invasion, N stage, T stage, tumor size, and grade. The importance of the features varied slightly across different machine learning models.

### The prediction of the risk of lung metastasis in patients with rectal cancer

**Figure 6:**
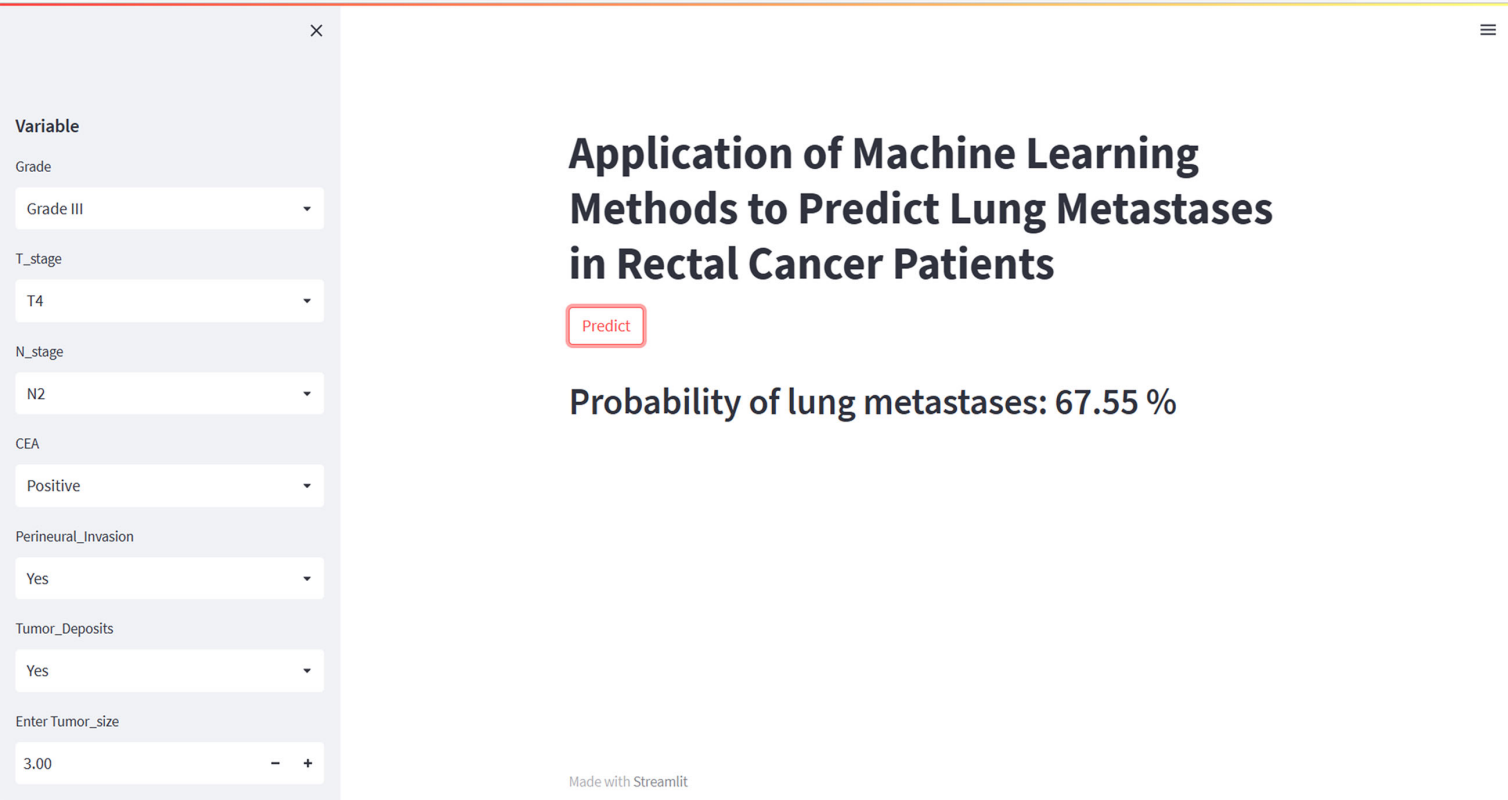
A web calculator for predicting lung metastases from rectal cancer.

To facilitate clinical use, we have developed an online web calculator based on the XGB model for predicting lung metastasis in rectal cancer. The XGB model showed superior predictive performance for rectal cancer lung metastasis, but its complexity makes it unsuitable for clinical application. The web calculator (https://share.streamlit.io/woshiwz/rectal_cancer/main/lung.py) enables users to input the patient’s clinicopathological information, and estimate the probability of lung metastasis occurrence in rectal cancer patients. **Figure 6** shows screenshots of the web calculator.

## Discussion

Timely detection and intervention of lung metastases are crucial in rectal cancer patients as they significantly predict poor prognosis. Early diagnosis of lung metastases can improve the feasibility of surgical treatment and overall survival. For instance, Heinemann et al. demonstrated that early detection of lung metastases for surgery improved the 5-year survival rate by 30% compared to patients who did not undergo surgery (7). However, lung metastases incidence may have been underestimated due to the lack of symptoms at the time of initial diagnosis or delayed presentation. Therefore, detecting lung metastases from rectal cancer at an early stage is challenging. Moreover, patients with lung metastases from rectal cancer may experience a reduced quality of life due to respiratory symptoms such as chest pain, coughing up blood, and respiratory failure (36). Although Positron Emission Tomography–Computed Tomography (PET-CT) is commonly used to diagnose lung metastases, its high cost and potential risk of radiological damage may make it unsuitable for early screening (37). The biopsy is the gold standard for diagnosing lung metastases, but the procedure’s complexity and the risk of tumor cell dissemination make it unsuitable for routine diagnosis (38). Given these limitations, this study utilized machine learning techniques based on clinicopathological indicators to develop predictive models to identify high-risk patients. These models could help clinicians develop personalized treatment plans for patients with rectal cancer, including asymptomatic patients with lung metastases.

In several studies, tumor size is an independent risk factor for cancer metastasis (39–43). The results of this study’s multivariable logistic regression and machine learning models are consistent with them. The larger the tumor size, the longer the tumor growth time, allowing for better tumor cell evolution and thus contributing to lung metastasis development. In a prospective controlled study involving 167 individuals, patients with lymph node metastases were more likely to have recurrence and distal metastases (44). This is because the main route of distal metastasis is lymphatic, and the lung is one of the most lymphatic-rich organs, where tumor cells are more likely to colonize the lung *via* the lymphatics. Not surprisingly, more advanced T-stage rectal cancers are more likely to develop lung metastases (39, 45). This is because the late T stage and tumor invasion of connective tissue contributes to tumor metastasis *via* blood vessels or lymphatic vessels. Numerous studies have shown that CEA is an essential indicator of recurrence and metastasis in patients with colorectal cancer (46, 47). Li et al. suggested that perineural invasion is an independent predictor of distal metastasis in rectal cancer, and our findings support their view (48). Some current studies suggest that cancer cells may metastasize to other body parts along the peripheral nervous system in the case of nerve invasion. However, few have explained why patients with perineural invasion are prone to distal metastasis, and further in-depth studies in this area are needed in the future. Tumor deposits were identified as a risk factor for lung metastasis from rectal cancer. These deposits refer to nodules of tumor tissue found within the lymphatic drainage area of the primary tumor, lacking lymph node, vascular, and nerve tissue, and can vary in size, shape, and border (49). Romian et al. showed that tumor deposits in colorectal cancer patients increased the risk of death by 59% and found that the prognosis of patients with tumor deposits was the same as that of patients with N1 lymph node metastases from rectal cancer (50).

To our knowledge, the study represents the first instance of utilizing machine learning algorithms with real-world data to predict lung metastasis in rectal cancer. In order to enhance the model’s versatility, we incorporated multicenter data from the SEER database and performed internal validation to confirm its reliability. Due to the heterogeneity of the data, we employed external validation to evaluate the model’s performance. Using the XGB algorithm, our study produced a machine-learning model for lung metastasis prediction in rectal cancer that outperformed other algorithms. The XGB algorithm demonstrated excellent predictive ability in training and validation cohorts. This algorithm’s superiority in managing large and non-linear datasets may be due to incorporating standard terms into the objective function to prevent overfitting and using column sampling to bolster model stability (46). Our study’s lung metastasis model for rectal cancer can give clinicians and patients a more precise estimate of the likelihood of lung metastasis in the clinical setting. Shortening the examination cycle for high-risk patients allows early detection and treatment of lung metastases from rectal cancer, thus improving patient prognosis and elevating their quality of life.

Although our developed model shows strong discriminatory power, this study has some drawbacks. Firstly, this is a retrospective study, and there may be a selection bias in the patient selection that needs to be validated in further prospective studies. Secondly, the external validation cohort was single-center data with a small number of patients who were all Asian. Therefore, more patient data from multiple hospitals will be needed to validate our model’s diagnostic efficacy and extrapolation. Thirdly, the absence of important information, including immunohistochemical information, hematologic indicators, and radiotherapy information due to data limitations in the SEER database, limited our ability to optimize the model further.

## Conclusion

In summary, we developed and validated a clinical prediction model for lung metastases from rectal cancer built on machine learning algorithms. We have created a visual web calculator based on the XGB algorithm, which helps doctors to individualize the treatment of patients at risk of lung metastasis from rectal cancer. In the future, we will further validate the model using multicenter data and evaluate its performance.

## Data availability statement

The original contributions presented in the study are included in the article/**Supplementary Material**. Further inquiries can be directed to the corresponding author.

## Ethics statement

Written informed consent was not obtained from the individual(s) for the publication of any potentially identifiable images or data included in this article.

## Author contributions

BQ and QW designed the study. BQ, ZS, and DY conducted data analysis. BQ conceived the project and wrote the manuscript. QW revised and approved the paper. All authors contributed to the article and approved the submitted version.
