# Supplementary material for: Applying machine learning techniques to predict the risk of lung metastases from rectal cancer: a real-world retrospective study
Source: Front Oncol. 2023 May 24;13:1183072. doi: 10.3389/fonc.2023.1183072 (PMC10247137; doi:10.3389/fonc.2023.1183072)
Supplement: Supplementary file 3 [file Table_3.docx]

import requests

import numpy as np

import pandas as pd

import sys

import xgboost

import matplotlib.pyplot as plt

from numpy import interp

import matplotlib.pyplot as set_facecolor

from xgboost import XGBClassifier

from sklearn.ensemble import RandomForestClassifier

from sklearn.neural_network import MLPClassifier

from sklearn import tree #导入需要的模块

from sklearn.ensemble import GradientBoostingClassifier

from sklearn.linear_model import LogisticRegression

from sklearn.tree import DecisionTreeClassifier

from sklearn.neighbors import KNeighborsClassifier

from catboost import CatBoostClassifier

import lightgbm as lgb

from sklearn.svm import SVC

from sklearn.model_selection import train_test_split, GroupKFold, KFold,StratifiedShuffleSplit, GridSearchCV, cross_val_score,StratifiedKFold

from sklearn.metrics import accuracy_score

import matplotlib

import scipy.stats as stats

from sklearn import metrics

from sklearn.tree import export_graphviz

from sklearn.metrics import roc_auc_score,roc_curve,auc

from sklearn.model_selection import cross_val_score as CVS

import sklearn.metrics as metrics

from sklearn.metrics import mean_squared_error as MSE

from sklearn.model_selection import GridSearchCV

from sklearn.model_selection import RandomizedSearchCV

from sklearn.model_selection import cross_validate

from sklearn.metrics import mean_absolute_error as mae

from sklearn.preprocessing import RobustScaler, normalize

from IPython.display import display

import seaborn as sns

from sklearn.cluster import KMeans

from sklearn.preprocessing import MinMaxScaler

from sklearn import svm

from sklearn.metrics import confusion_matrix

import eli5

from eli5.sklearn import PermutationImportance

from IPython.display import display, Image

import shap

from sklearn.metrics import classification_report

import webbrowser

from sklearn.metrics import precision_recall_curve,precision_score,recall_score,average_precision_score,ConfusionMatrixDisplay

from sklearn.naive_bayes import GaussianNB,MultinomialNB

from imblearn import over_sampling

from imblearn.over_sampling import SMOTE,RandomOverSampler

from collections import Counter

import scikitplot as skp

from sklearn.preprocessing import MinMaxScaler,StandardScaler

##加载数据

data=pd.read_csv("E:\\SEER肺转移\\data3.csv")

y= data.Lung_Met#因子变量

X = data.drop('Lung_Met',1)

data2=pd.read_csv("E:\\SEER肺转移\\data4.csv")

Yvalid= data2.Lung_Met#因子变量

Xvalid = data2.drop('Lung_Met',1)

##

###

Xtrain,Xtest,Ytrain,Ytest = train_test_split(X,y,test_size=0.3,random_state=420)

Xtest.to_csv(r'E:/SEER肺转移/Xtest.csv')

Ytest.to_csv(r'E:/SEER肺转移/Ytest.csv')

Xtrain.to_csv(r'E:/SEER肺转移/Xtrain.csv')

Ytrain.to_csv(r'E:/SEER肺转移/Ytrain.csv')

Xvalid.to_csv(r'E:/SEER肺转移/Xvalid.csv')

Yvalid.to_csv(r'E:/SEER肺转移/Yvalid.csv')

##对数据进行过采样

sos = SMOTE(random_state=0,sampling_strategy=0.3)

Xtrain,Ytrain= sos.fit_resample(Xtrain,Ytrain)

####

Xtrain=pd.DataFrame(Xtrain)

Xtest=pd.DataFrame(Xtest)

Xvalid=pd.DataFrame(Xvalid)

Xtrain.columns=['Grade','T_stage','N_stage',"CEA",'PI','Tumor_deposit',"Tumor_size"]

Xtest.columns=['Grade','T_stage','N_stage',"CEA",'PI','Tumor_deposit',"Tumor_size"]

Xvalid.columns=['Grade','T_stage','N_stage',"CEA",'PI','Tumor_deposit',"Tumor_size"]

f_top='Grade+T_stage+N_stage+CEA+PI+Tumor_deposit+Tumor_size'

f_top=f_top.split('+')

f_top

##

modelRF=RandomForestClassifier(n_estimators=200,max_depth=100,min_samples_leaf=2,min_samples_split=2)

modelKNN=KNeighborsClassifier(n_neighbors=1)

modelLR=LogisticRegression(penalty='l2',random_state =1,C=0.1)

modelMLP=MLPClassifier()

modelSVM=SVC(probability=(True))

modelDT= DecisionTreeClassifier(max_depth = 100,criterion='gini',

min_samples_leaf=1,min_samples_split=2,random_state = 1)

modelBNB= GaussianNB(var_smoothing=100)

modelXGB= XGBClassifier(

booster='gbtree',

objective='binary:logistic',eval_metric=['auc'],

max_depth=120,

n_estimators=200,#300

min_child_weight=1,

learning_rate=0.08,

random_state=0,

gamma=0.8,

reg_lambda=1,

reg_alpha=1,

scale_pos_weight=1,

subsample=0.8,

colsample_bytree=0.8,

seed=1,

n_jobs=-1)

##

##

n_estimators = [1,200,10]

max_depth = [1,120,10]

scale_pos_weight=[1,5,1]

random_grid = {'n_estimators': n_estimators,

'max_depth': max_depth,

'scale_pos_weight':scale_pos_weight,

}

XGB_random = RandomizedSearchCV(estimator =modelXGB,

param_distributions = random_grid,

n_iter = 50, cv = 5, verbose=2,random_state=35, n_jobs = -1)

XGB_random.fit(Xtrain,Ytrain)

# this prints the contents of the parameters in the random grid

print ('Random grid: ', random_grid, '\n')

# print the best parameters

print ('Best Parameters: ', XGB_random.best_params_, ' \n')

##

n_estimators = [1,200,10]

max_depth = [1,100,10]

min_samples_leaf = [1, 2, 4]

random_grid = {

"n_estimators": n_estimators,

"max_depth": max_depth,

"min_samples_leaf": min_samples_leaf,

}

RF_random = RandomizedSearchCV(estimator =modelRF,

param_distributions = random_grid,

n_iter = 100, cv = 5, verbose=2, random_state=35, n_jobs = -1)

RF_random.fit(Xtrain,Ytrain)

# this prints the contents of the parameters in the random grid

print ('Random grid: ', random_grid, '\n')

# print the best parameters

print ('Best Parameters: ', RF_random.best_params_, ' \n')

##

random_grid = {'C': [0.1, 1, 10, 100],

'kernel': ['linear', 'rbf', 'poly', 'sigmoid'],

'gamma': ['scale', 'auto'],

'degree': [2, 3, 4, 5],

'coef0': [0, 1, 2, 3]}

svm_random = RandomizedSearchCV(estimator =modelSVM,

param_distributions = random_grid,

n_iter = 100, cv = 5, verbose=2, random_state=35, n_jobs = -1)

svm_random.fit(Xtrain,Ytrain)

# this prints the contents of the parameters in the random grid

print ('Random grid: ', random_grid, '\n')

# print the best parameters

print ('Best Parameters: ',svm_random.best_params_, ' \n')

##

C=[0,0.1,0.001]

random_grid = {'C': C}

LR_random = RandomizedSearchCV(estimator =modelLR,

param_distributions = random_grid,

n_iter = 100, cv = 5, verbose=2, random_state=35, n_jobs = -1)

LR_random.fit(Xtrain,Ytrain)

# this prints the contents of the parameters in the random grid

print ('Random grid: ', random_grid, '\n')

# print the best parameters

print ('Best Parameters: ',LR_random.best_params_, ' \n')

##

n_neighbors=[1,200,2]

random_grid = {'n_neighbors':n_neighbors}

KNN_random = RandomizedSearchCV(estimator =modelKNN,

param_distributions = random_grid,

n_iter = 100, cv = 5, verbose=2, random_state=35, n_jobs = -1)

KNN_random.fit(Xtrain,Ytrain)

# this prints the contents of the parameters in the random grid

print ('Random grid: ', random_grid, '\n')

# print the best parameters

print ('Best Parameters: ',KNN_random.best_params_, ' \n')

##

max_depth = [1,100,10]

min_samples_split = [1,8,2]

min_samples_leaf = [1, 2, 4]

random_grid = {

"max_depth": max_depth,

"min_samples_split": min_samples_split,

"min_samples_leaf": min_samples_leaf,

}

RF_random = RandomizedSearchCV(estimator =modelRF,

param_distributions = random_grid,

n_iter = 100, cv = 5, verbose=2, random_state=35, n_jobs = -1)

RF_random.fit(Xtrain,Ytrain)

# this prints the contents of the parameters in the random grid

print ('Random grid: ', random_grid, '\n')

# print the best parameters

print ('Best Parameters: ', RF_random.best_params_, ' \n')

#####

##

modelXGB=modelXGB.fit(Xtrain,Ytrain)

modelRF=modelRF.fit(Xtrain,Ytrain)

modelKNN=modelKNN.fit(Xtrain,Ytrain)

modelLR=modelLR.fit(Xtrain,Ytrain)

modelDT=modelDT.fit(Xtrain,Ytrain)

modelMLP=modelMLP.fit(Xtrain,Ytrain)

modelSVM=modelSVM.fit(Xtrain,Ytrain)

modelBNB=modelBNB.fit(Xtrain,Ytrain)

##

##

result1=[]

for model in [modelXGB,modelRF,modelKNN,modelLR,modelDT,modelMLP,modelSVM,modelBNB]:

result1.append([round(metrics.roc_auc_score(Ytrain,model.predict_proba(Xtrain[f_top])[:,1]),2)

,round(metrics.accuracy_score(Ytrain,model.predict(Xtrain[f_top])),2)

,float(metrics.classification_report(Ytrain,model.predict(Xtrain[f_top])).split('\n')[-2].split(' ')[1].replace(' ',''))

,float(metrics.classification_report(Ytrain,model.predict(Xtrain[f_top])).split('\n')[-2].split(' ')[2].replace(' ',''))

,float(metrics.classification_report(Ytrain,model.predict(Xtrain[f_top])).split('\n')[-2].split(' ')[3].replace(' ',''))])

result1=pd.DataFrame(result1,columns=['AUC','Accuracy','precision','recall','f1-score'],index=['XGB','DT','RF','LR','MLP','NBC','SVM','KNN'])

result2=[]

for model in [modelXGB,modelRF,modelKNN,modelLR,modelDT,modelMLP,modelSVM,modelBNB]:

result2.append([round(metrics.roc_auc_score(Ytest,model.predict_proba(Xtest[f_top])[:,1]),2)

,round(metrics.accuracy_score(Ytest,model.predict(Xtest[f_top])),2)

,float(metrics.classification_report(Ytest,model.predict(Xtest[f_top])).split('\n')[-2].split(' ')[1].replace(' ',''))

,float(metrics.classification_report(Ytest,model.predict(Xtest[f_top])).split('\n')[-2].split(' ')[2].replace(' ',''))

,float(metrics.classification_report(Ytest,model.predict(Xtest[f_top])).split('\n')[-2].split(' ')[3].replace(' ',''))])

result2=pd.DataFrame(result1,columns=['AUC','Accuracy','precision','recall','f1-score'],index=['XGB','DT','RF','LR','MLP','NBC','SVM','KNN'])

result3=[]

for model in [modelXGB,modelRF,modelKNN,modelLR,modelDT,modelMLP,modelSVM,modelBNB]:

result3.append([round(metrics.roc_auc_score(Yvalid,model.predict_proba(Xvalid)[:,1]),2)

,round(metrics.accuracy_score(Yvalid,model.predict(Xvalid)),2)

,float(metrics.classification_report(Yvalid,model.predict(Xvalid)).split('\n')[-2].split(' ')[1].replace(' ',''))

,float(metrics.classification_report(Yvalid,model.predict(Xvalid)).split('\n')[-2].split(' ')[2].replace(' ',''))

,float(metrics.classification_report(Yvalid,model.predict(Xvalid)).split('\n')[-2].split(' ')[3].replace(' ',''))])

result3=pd.DataFrame(result3,columns=['AUC','Accuracy','precision','recall','f1-score'],index=['XGB','DT','RF','LR','MLP','NBC','SVM','KNN'])

fig = plt.gcf()

fig.set_size_inches(20,12)

plt.subplot(1,3,1)

sns.heatmap(data=result1,

vmax=1,

vmin=0.6,

cmap='YlOrRd_r',

annot=True,

fmt=".3f",

)

plt.xticks(fontsize=20,rotation=60)

plt.yticks(fontsize=20,rotation=0)

plt.title('train set')

plt.subplot(1,3,2)

sns.heatmap(data=result2,

vmax=1,

vmin=0.6,

cmap='YlOrRd_r',

annot=True,

fmt=".3f",

)

plt.xticks(fontsize=20,rotation=60)

plt.yticks(fontsize=20,rotation=0)

plt.title('internal test set')

plt.subplot(1,3,3)

sns.heatmap(data=result3,

vmax=1,

vmin=0.6,

cmap='YlOrRd_r',

annot=True,

fmt=".3f",

)

plt.xticks(fontsize=20,rotation=60)

plt.yticks(fontsize=20,rotation=0)

plt.title('external test set')

plt.savefig('热力图.tiff',dpi=300,bbox_inches = 'tight')

###################################################################

###################################################################

###################################################################

###################################################################

###################################################################

##十倍交叉验证

from sklearn.model_selection import cross_val_score,StratifiedKFold,LeaveOneOut

strKFold = StratifiedKFold(n_splits=10,shuffle=True,random_state=11)

# loout = LeaveOneOut()

cv=strKFold

# cv=loout

result_LR=cross_val_score(modelLR,Xtrain[f_top],Ytrain,scoring='roc_auc',cv=cv,n_jobs=-1)

result_XGB=cross_val_score(modelXGB,Xtrain[f_top],Ytrain,scoring='roc_auc',cv=cv,n_jobs=-1)

result_BNB=cross_val_score(modelBNB,Xtrain[f_top],Ytrain,scoring='roc_auc',cv=cv,n_jobs=-1)

result_RF=cross_val_score(modelRF,Xtrain[f_top],Ytrain,scoring='roc_auc',cv=cv,n_jobs=-1)

result_MLP=cross_val_score(modelMLP,Xtrain[f_top],Ytrain,scoring='roc_auc',cv=cv,n_jobs=-1)

result_KNN=cross_val_score(modelKNN,Xtrain[f_top],Ytrain,scoring='roc_auc',cv=cv,n_jobs=-1)

result_SVM=cross_val_score(modelSVM,Xtrain[f_top],Ytrain,scoring='roc_auc',cv=cv,n_jobs=-1)

result_DT=cross_val_score(modelDT,Xtrain[f_top],Ytrain,scoring='roc_auc',cv=cv,n_jobs=-1)

fig = plt.gcf()

fig.set_size_inches(15,8)

n=10

plt.plot(range(n),result_LR, marker=">", ms=12,label='LR Average AUC=%s,Std=%s'%(round(result_LR.mean(),2),round(result_LR.std(),2)),color='red')

plt.plot(range(n),result_XGB, marker=">", ms=12,label='XGB Average AUC=%s,Std=%s'%(round(result_XGB.mean(),2),round(result_XGB.std(),2)),color='blue')

plt.plot(range(n),result_BNB, marker=">", ms=12,label='BNB Average AUC=%s,Std=%s'%(round(result_BNB.mean(),2),round(result_BNB.std(),2)),color='m')

plt.plot(range(n),result_RF, marker=">", ms=12,label='RF Average AUC=%s,Std=%s'%(round(result_RF.mean(),2),round(result_RF.std(),2)),color='green')

plt.plot(range(n),result_MLP, marker=">", ms=12,label='MLP Average AUC=%s,Std=%s'%(round(result_MLP.mean(),2),round(result_MLP.std(),2)),color='tomato')

plt.plot(range(n),result_KNN, marker=">", ms=12,label='KNN Average AUC=%s,Std=%s'%(round(result_KNN.mean(),2),round(result_KNN.std(),2)),color='darkblue')

plt.plot(range(n),result_SVM, marker=">", ms=12,label='SVM Average AUC=%s,Std=%s'%(round(result_SVM.mean(),2),round(result_SVM.std(),2)),color='deepskyblue')

plt.plot(range(n),result_DT, marker=">", ms=12,label='DT Average AUC=%s,Std=%s'%(round(result_DT.mean(),2),round(result_DT.std(),2)),color='gray')

plt.legend(loc=4)

plt.ylim(0,1)

plt.savefig('交叉验证.tiff',dpi=600)

###################################################################

###################################################################

###################################################################

###################################################################

###################################################################

##

plt.style.use('tableau-colorblind10')

def plot_roc(k,y_pred_undersample_score,labels_test,classifiers,color,title):

fpr, tpr, thresholds = metrics.roc_curve(labels_test.values.ravel(),y_pred_undersample_score)

roc_auc = metrics.auc(fpr,tpr)

plt.figure(figsize=(20,16))

plt.figure(k)

plt.title(title)

plt.plot(fpr, tpr, 'b',color=color,label='%s AUC = %0.2f'% (classifiers,roc_auc))

plt.legend(loc='lower right')

plt.plot([0,1],[0,1],'r--')

plt.xlim([-0.1,1.0])

plt.ylim([-0.1,1.01])

plt.ylabel('True Positive Rate')

plt.xlabel('False Positive Rate')

fig = plt.gcf()

fig.set_size_inches(8,8)

# plt.subplot(1,3,1)

plot_roc(1,modelLR.predict_proba(Xtrain)[:,1],Ytrain,'LR','red','train ROC curve')

plot_roc(1,modelXGB.predict_proba(Xtrain)[:,1],Ytrain,'XGB','blue','train ROC curve')

plot_roc(1,modelBNB.predict_proba(Xtrain)[:,1],Ytrain,'BNB','m','train ROC curve')

plot_roc(1,modelRF.predict_proba(Xtrain)[:,1],Ytrain,'RF','green','train ROC curve')

plot_roc(1,modelMLP.predict_proba(Xtrain)[:,1],Ytrain,'MLP','tomato','train ROC curve')

plot_roc(1,modelKNN.predict_proba(Xtrain)[:,1],Ytrain,'KNN','darkblue','train ROC curve')

plot_roc(1,modelSVM.predict_proba(Xtrain)[:,1],Ytrain,'SVM','deepskyblue','train ROC curve')

plot_roc(1,modelBNB.predict_proba(Xtrain)[:,1],Ytrain,'BNB','pink','train ROC curve')

plt.savefig('TrainROC.tiff',dpi=600)

# plt.subplot(1,3,2)

fig = plt.gcf()

fig.set_size_inches(8,8)

plot_roc(1,modelLR.predict_proba(Xtest)[:,1],Ytest,'LR','red','test ROC curve')

plot_roc(1,modelXGB.predict_proba(Xtest)[:,1],Ytest,'XGB','blue','test ROC curve')

plot_roc(1,modelBNB.predict_proba(Xtest)[:,1],Ytest,'BNB','m','test ROC curve')

plot_roc(1,modelRF.predict_proba(Xtest)[:,1],Ytest,'RF','green','test ROC curve')

plot_roc(1,modelMLP.predict_proba(Xtest)[:,1],Ytest,'MLP','tomato','test ROC curve')

plot_roc(1,modelKNN.predict_proba(Xtest)[:,1],Ytest,'KNN','darkblue','test ROC curve')

plot_roc(1,modelSVM.predict_proba(Xtest)[:,1],Ytest,'SVM','deepskyblue','test ROC curve')

plot_roc(1,modelBNB.predict_proba(Xtest)[:,1],Ytest,'BNB','pink','test ROC curve')

plt.savefig('TestROC.tiff',dpi=600)

# plt.subplot(1,3,3)

fig = plt.gcf()

fig.set_size_inches(8,8)

plot_roc(1,modelLR.predict_proba(Xvalid)[:,1],Yvalid,'LR','red','valid ROC curve')

plot_roc(1,modelXGB.predict_proba(Xvalid)[:,1],Yvalid,'XGB','blue','valid ROC curve')

plot_roc(1,modelBNB.predict_proba(Xvalid)[:,1],Yvalid,'BNB','m','valid ROC curve')

plot_roc(1,modelRF.predict_proba(Xvalid)[:,1],Yvalid,'RF','green','valid ROC curve')

plot_roc(1,modelMLP.predict_proba(Xvalid)[:,1],Yvalid,'MLP','tomato','valid ROC curve')

plot_roc(1,modelKNN.predict_proba(Xvalid)[:,1],Yvalid,'KNN','darkblue','valid ROC curve')

plot_roc(1,modelSVM.predict_proba(Xvalid)[:,1],Yvalid,'SVM','deepskyblue','valid ROC curve')

plot_roc(1,modelDT.predict_proba(Xvalid)[:,1],Yvalid,'DT','deepskyblue','valid ROC curve')

plt.savefig('ValidROC.tiff',dpi=600)

###################################################################

###################################################################

###################################################################

###################################################################

###################################################################

###

#

from sklearn.svm import SVC

from sklearn.metrics import precision_recall_curve, auc

from sklearn.ensemble import VotingClassifier

classifiers = [

RandomForestClassifier(n_estimators=200,max_depth=100,min_samples_leaf=2,min_samples_split=2),

KNeighborsClassifier(n_neighbors=1),

LogisticRegression(penalty='l2',random_state =1,C=0.1),

MLPClassifier(),

SVC(probability=True),

DecisionTreeClassifier(max_depth = 100,criterion='gini',min_samples_leaf=1,min_samples_split=2,random_state = 1),

GaussianNB(var_smoothing=100),

XGBClassifier( booster='gbtree',

objective='binary:logistic',eval_metric=['auc'],

max_depth=120,

n_estimators=200,#300

min_child_weight=1,

learning_rate=0.08,

random_state=0,

gamma=0.8,

reg_lambda=1,

reg_alpha=1,

scale_pos_weight=1,

subsample=0.8,

colsample_bytree=0.8,

seed=1,

n_jobs=-1)]

fig = plt.gcf()

fig.set_size_inches(8,8)

names = ['RF', 'KNN', 'LR', 'MLP',"SVM","DT",'BNB',"XGB",]

# plt.subplot(1,3,1)

for clf, name in zip(classifiers, names):

clf.fit(Xtrain, Ytrain)

y_scores = clf.predict_proba(Xtrain)[:, 1]

precision, recall, thresholds = precision_recall_curve(Ytrain, y_scores)

pr_auc = auc(recall, precision)

plt.plot(recall, precision, label='{} (PR = {:.3f})'.format(name, pr_auc))

# Plot the PR curve

plt.xlabel('Recall')

plt.ylabel('Precision')

plt.title('Precision-Recall Curves of Different Classifiers')

plt.legend(loc='lower right',fontsize=8)

plt.savefig('TrainPR.tiff',dpi=600)

#

fig = plt.gcf()

fig.set_size_inches(8,8)

names = ['RF', 'KNN', 'LR', 'MLP',"SVM","DT",'BNB','XGB']

# plt.subplot(1,3,2)

for clf, name in zip(classifiers, names):

clf.fit(Xtrain, Ytrain)

y_scores = clf.predict_proba(Xtest)[:, 1]

precision, recall, thresholds = precision_recall_curve(Ytest, y_scores)

pr_auc = auc(recall, precision)

plt.plot(recall, precision, label='{} (PR = {:.3f})'.format(name, pr_auc))

# Plot the PR curve

plt.xlabel('Recall')

plt.ylabel('Precision')

plt.title('Precision-Recall Curves of Different Classifiers')

plt.legend(loc='lower right',fontsize=8)

plt.savefig('TestPR.tiff',dpi=600)

#

fig = plt.gcf()

fig.set_size_inches(8,8)

# plt.subplot(1,3,3)

for clf, name in zip(classifiers, names):

clf.fit(Xtrain, Ytrain)

y_scores = clf.predict_proba(Xvalid)[:, 1]

precision, recall, thresholds = precision_recall_curve(Yvalid, y_scores)

pr_auc = auc(recall, precision)

plt.plot(recall, precision, label='{} (PR = {:.3f})'.format(name, pr_auc))

# Plot the PR curve

plt.xlabel('Recall')

plt.ylabel('Precision')

plt.title('Precision-Recall Curves of Different Classifiers')

plt.legend(loc='lower right',fontsize=8)

plt.savefig('ValidPR.tiff',dpi=600)

###################################################################

###################################################################

###################################################################

###################################################################

###################################################################

#

from sklearn import preprocessing

def dac(pred_ans,train,f_top,k,color,name,title,aaa=0.05):

Y = Ytrain

a=Ytrain.value_counts()[0]

b=Ytrain.value_counts()[1]

pt_arr = []

net_bnf_arr = []

jiduan = []

pred_ans = pred_ans.ravel()

for i in range(0,100,1):

pt = i /100

#compiute TP FP

pred_ans_clip = np.zeros(pred_ans.shape[0])

for j in range(pred_ans.shape[0]):

if pred_ans[j] >= pt:

pred_ans_clip[j] = 1

else:

pred_ans_clip[j] = 0

TP = np.sum((Y) * np.round(pred_ans_clip))

FP = np.sum((1 - Y) * np.round(pred_ans_clip))

net_bnf = ( TP-(FP * pt/(1-pt)) )/Y.shape[0]

# print('pt {}, TP {}, FP {}, net_bf {}'.format(pt,TP,FP,net_bnf))

pt_arr.append(pt)

net_bnf_arr.append(net_bnf)

jiduan.append((b-a*pt/(1-pt))/(a+b))

plt.figure(figsize=(12,8))

plt.figure(k)

plt.plot(pt_arr, net_bnf_arr, color=color, lw=2,label=name)

plt.legend(loc=4)

plt.plot(pt_arr, np.zeros(len(pt_arr)), color='k', lw=2)

# ,label='None'

# data_test = data_test.ravel()

pt_np = np.array(pt_arr)

# jiduan = (np.sum(data_test)-(len(data_test)-np.sum(data_test)*pt_np)/(1-pt_np))/len(data_test)

plt.plot(pt_arr, jiduan , color='b', lw=2, linestyle='dotted')

# ,label='ALL'

plt.xlim([0.0, 1.0])

plt.ylim([-0.06, 0.1])

plt.xlabel('Risk Threshold')

plt.ylabel('Net Benefit')

plt.title(title)

# plt.savefig("DCA.png")

# plt.show()

fig = plt.gcf()

fig.set_size_inches(8,8)

# plt.subplot(1,3,1)

dac(modelLR.predict_proba(Xtrain[f_top])[:,1],Xtrain,f_top,1,'red','LR','Train data')

dac(modelXGB.predict_proba(Xtrain[f_top])[:,1],Xtrain,f_top,1,'deepskyblue','DT','Train data')

dac(modelMLP.predict_proba(Xtrain[f_top])[:,1],Xtrain,f_top,1,'m','MLP','Train data')

dac(modelBNB.predict_proba(Xtrain[f_top])[:,1],Xtrain,f_top,1,'green','BNB','Train data')

dac(modelRF.predict_proba(Xtrain[f_top])[:,1],Xtrain,f_top,1,'tomato','RF','Train data')

dac(modelKNN.predict_proba(Xtrain[f_top])[:,1],Xtrain,f_top,1,'darkblue','KNN','Train data')

dac(modelSVM.predict_proba(Xtrain[f_top])[:,1],Xtrain,f_top,1,'deepskyblue','SVM','Train data')

dac(modelDT.predict_proba(Xtrain[f_top])[:,1],Xtrain,f_top,1,'blue','XGB','Train data')

plt.savefig('TrainDCA.tiff',dpi=600)

##

def dac(pred_ans,train,f_top,k,color,name,title,aaa=0.05):

Y = Ytest

a=Ytest.value_counts()[0]

b=Ytest.value_counts()[1]

pt_arr = []

net_bnf_arr = []

jiduan = []

pred_ans = pred_ans.ravel()

for i in range(0,100,1):

pt = i /100

#compiute TP FP

pred_ans_clip = np.zeros(pred_ans.shape[0])

for j in range(pred_ans.shape[0]):

if pred_ans[j] >= pt:

pred_ans_clip[j] = 1

else:

pred_ans_clip[j] = 0

TP = np.sum((Y) * np.round(pred_ans_clip))

FP = np.sum((1 - Y) * np.round(pred_ans_clip))

net_bnf = ( TP-(FP * pt/(1-pt)) )/Y.shape[0]

# print('pt {}, TP {}, FP {}, net_bf {}'.format(pt,TP,FP,net_bnf))

pt_arr.append(pt)

net_bnf_arr.append(net_bnf)

jiduan.append((b-a*pt/(1-pt))/(a+b))

plt.figure(figsize=(12,8))

plt.figure(k)

plt.plot(pt_arr, net_bnf_arr, color=color, lw=2,label=name)

plt.legend(loc=4)

plt.plot(pt_arr, np.zeros(len(pt_arr)), color='k', lw=2)

# ,label='None'

# data_test = data_test.ravel()

pt_np = np.array(pt_arr)

# jiduan = (np.sum(data_test)-(len(data_test)-np.sum(data_test)*pt_np)/(1-pt_np))/len(data_test)

plt.plot(pt_arr, jiduan , color='b', lw=2, linestyle='dotted')

# ,label='ALL'

plt.xlim([0.0, 1.0])

plt.ylim([-0.06, 0.1])

plt.xlabel('Risk Threshold')

plt.ylabel('Net Benefit')

plt.title(title)

# plt.savefig("DCA.png")

# plt.show()

fig = plt.gcf()

fig.set_size_inches(8,8)

# plt.subplot(1,3,2)

dac(modelLR.predict_proba(Xtest[f_top])[:,1],Xtest,f_top,1,'red','LR','Test data')

dac(modelXGB.predict_proba(Xtest[f_top])[:,1],Xtest,f_top,1,'blue','XGB','Test data')

dac(modelMLP.predict_proba(Xtest[f_top])[:,1],Xtest,f_top,1,'m','MLP','Test data')

dac(modelBNB.predict_proba(Xtest[f_top])[:,1],Xtest,f_top,1,'green','BNB','Test data')

dac(modelRF.predict_proba(Xtest[f_top])[:,1],Xtest,f_top,1,'tomato','RF','Test data')

dac(modelKNN.predict_proba(Xtest[f_top])[:,1],Xtest,f_top,1,'darkblue','KNN','Test data')

dac(modelSVM.predict_proba(Xtest[f_top])[:,1],Xtest,f_top,1,'deepskyblue','SVM','Test data')

dac(modelDT.predict_proba(Xtest[f_top])[:,1],Xtest,f_top,1,'deepskyblue','DT','Test data')

plt.savefig('TestDCA.tiff',dpi=600)

#

def dac(pred_ans,train,f_top,k,color,name,title,aaa=0.05):

Y = Yvalid

a=Yvalid.value_counts()[0]

b=Yvalid.value_counts()[1]

pt_arr = []

net_bnf_arr = []

jiduan = []

pred_ans = pred_ans.ravel()

for i in range(0,100,1):

pt = i /100

#compiute TP FP

pred_ans_clip = np.zeros(pred_ans.shape[0])

for j in range(pred_ans.shape[0]):

if pred_ans[j] >= pt:

pred_ans_clip[j] = 1

else:

pred_ans_clip[j] = 0

TP = np.sum((Y) * np.round(pred_ans_clip))

FP = np.sum((1 - Y) * np.round(pred_ans_clip))

net_bnf = ( TP-(FP * pt/(1-pt)) )/Y.shape[0]

# print('pt {}, TP {}, FP {}, net_bf {}'.format(pt,TP,FP,net_bnf))

pt_arr.append(pt)

net_bnf_arr.append(net_bnf)

jiduan.append((b-a*pt/(1-pt))/(a+b))

plt.figure(figsize=(12,8))

plt.figure(k)

plt.plot(pt_arr, net_bnf_arr, color=color, lw=2,label=name)

plt.legend(loc=4)

plt.plot(pt_arr, np.zeros(len(pt_arr)), color='k', lw=2)

# ,label='None'

# data_test = data_test.ravel()

pt_np = np.array(pt_arr)

# jiduan = (np.sum(data_test)-(len(data_test)-np.sum(data_test)*pt_np)/(1-pt_np))/len(data_test)

plt.plot(pt_arr, jiduan , color='b', lw=2, linestyle='dotted')

# ,label='ALL'

plt.xlim([0.0, 1.0])

plt.ylim([-0.06, 0.15])

plt.xlabel('Risk Threshold')

plt.ylabel('Net Benefit')

plt.title(title)

# plt.savefig("DCA.png")

# plt.show()

fig = plt.gcf()

fig.set_size_inches(8,8)

# plt.subplot(1,3,3)

dac(modelLR.predict_proba(Xvalid[f_top])[:,1],Yvalid,f_top,1,'red','LR','Valid data')

dac(modelXGB.predict_proba(Xvalid[f_top])[:,1],Yvalid,f_top,1,'blue','XGB','Valid data')

dac(modelMLP.predict_proba(Xvalid[f_top])[:,1],Yvalid,f_top,1,'m','MLP','Valid data')

dac(modelBNB.predict_proba(Xvalid[f_top])[:,1],Yvalid,f_top,1,'green','BNB','Valid data')

dac(modelRF.predict_proba(Xvalid[f_top])[:,1],Yvalid,f_top,1,'tomato','RF','Valid data')

dac(modelKNN.predict_proba(Xvalid[f_top])[:,1],Yvalid,f_top,1,'darkblue','KNN','Valid data')

dac(modelSVM.predict_proba(Xvalid[f_top])[:,1],Yvalid,f_top,1,'deepskyblue','SVM','Valid data')

dac(modelDT.predict_proba(Xvalid[f_top])[:,1],Yvalid,f_top,1,'deepskyblue','DT','Valid data')

plt.savefig('ValidDCA.tiff',dpi=600)

###################################################################

###################################################################

###################################################################

###################################################################

###################################################################

###

fig = plt.gcf()

fig.set_size_inches(8,8)

from sklearn import preprocessing

min_max_scaler1 = preprocessing.MinMaxScaler(feature_range = (0,100),copy = 1)

# plt.subplot(3,3,1)

tmp=pd.DataFrame([abs(modelLR.coef_[0]),f_top]).T.sort_values(by=0,ascending=False)

tmp.columns=['value','col']

tmp['value']=min_max_scaler1.fit_transform(tmp[['value']])

sns.barplot(x=tmp.value,y=tmp.col,palette='twilight_shifted')

plt.ylabel('')

plt.xlabel('')

plt.title('Feature Importances of LR')

plt.savefig('LR.tiff',dpi=300)

# plt.subplot(3,3,2)

fig = plt.gcf()

fig.set_size_inches(8,8)

tmp=pd.DataFrame([abs(modelRF.feature_importances_),f_top]).T.sort_values(by=0,ascending=False)

tmp.columns=['value','col']

tmp['value']=min_max_scaler1.fit_transform(tmp[['value']])

sns.barplot(x=tmp.value,y=tmp.col,palette='turbo_r')

plt.ylabel('')

plt.xlabel('')

plt.title('Feature Importances of RF')

plt.savefig('RF.tiff',dpi=300)

# plt.subplot(3,3,3)

fig = plt.gcf()

fig.set_size_inches(8,8)

tmp=pd.DataFrame([abs(modelXGB.feature_importances_),f_top]).T.sort_values(by=0,ascending=False)

tmp.columns=['value','col']

tmp['value']=min_max_scaler1.fit_transform(tmp[['value']])

sns.barplot(x=tmp.value,y=tmp.col,palette='winter')

plt.ylabel('')

plt.xlabel('')

plt.title('Feature Importances of XGB')

plt.savefig('XGB.tiff',dpi=300)

# plt.subplot(3,3,4)

fig = plt.gcf()

fig.set_size_inches(8,8)

tmp=pd.DataFrame([abs(modelDT.feature_importances_),f_top]).T.sort_values(by=0,ascending=False)

tmp.columns=['value','col']

tmp['value']=min_max_scaler1.fit_transform(tmp[['value']])

sns.barplot(x=tmp.value,y=tmp.col,palette='winter')

plt.ylabel('')

plt.xlabel('')

plt.title('Feature Importances of DT')

plt.savefig('DT.tiff',dpi=300)

# plt.subplot(3,3,5)

fig = plt.gcf()

fig.set_size_inches(8,8)

tmp=pd.DataFrame([abs(np.dot(modelMLP.coefs_[0],modelMLP.coefs_[1]).reshape(1,-1)[0]),f_top]).T.sort_values(by=0,ascending=False)

tmp.columns=['value','col']

tmp['value']=min_max_scaler1.fit_transform(tmp[['value']])

sns.barplot(x=tmp.value,y=tmp.col,palette='gist_stern')

plt.ylabel('')

plt.xlabel('')

plt.title('Feature Importances of MLP')

plt.savefig('MLP.tiff',dpi=300)

# plt.subplot(3,3,6)

fig = plt.gcf()

fig.set_size_inches(8,8)

tmp=pd.DataFrame([abs(modelBNB.sigma_.mean(axis=0)),f_top]).T.sort_values(by=0,ascending=False)

tmp.columns=['value','col']

tmp['value']=min_max_scaler1.fit_transform(tmp[['value']])

sns.barplot(x=tmp.value,y=tmp.col,palette='coolwarm')

plt.ylabel('')

plt.xlabel('')

plt.title('Feature Importances of NBC')

plt.savefig('NBC.tiff',dpi=300)

# plt.subplot(3,3,7)

fig = plt.gcf()

fig.set_size_inches(8,8)

tmp=pd.DataFrame([abs(modelSVM.feature_importances_),f_top]).T.sort_values(by=0,ascending=False)

tmp.columns=['value','col']

tmp['value']=min_max_scaler1.fit_transform(tmp[['value']])

sns.barplot(x=tmp.value,y=tmp.col,palette='CMRmap_r')

plt.ylabel('')

plt.xlabel('')

plt.title('Feature Importances of svm')

plt.savefig('变量重要性.tiff',dpi=300)

perm = PermutationImportance(modelSVM, random_state=1).fit(Xtrain, Ytrain)

html_obj=eli5.show_weights(perm, feature_names = Xtrain.columns.tolist(),top=7)

with open('C:\\Users\qiubinxu\iris-importance.htm','wb') as f:

f.write(html_obj.data.encode("UTF-8"))

# Open the stored HTML file on the default browser

url = r'C:\\Users\qiubinxu\iris-importance.htm'

webbrowser.open(url, new=2)

perm = PermutationImportance(modelKNN, random_state=1).fit(Xtrain, Ytrain)

html_obj=eli5.show_weights(perm, feature_names = Xtrain.columns.tolist(),top=7)

with open('C:\\Users\qiubinxu\iris-importance.htm','wb') as f:

f.write(html_obj.data.encode("UTF-8"))

# Open the stored HTML file on the default browser

url = r'C:\\Users\qiubinxu\iris-importance.htm'

webbrowser.open(url, new=2)

fig = plt.gcf()

fig.set_size_inches(8,8)

labels = ['Grade','N-stage','PI','Tumor Deposits','CEA','T-stage','Tumor.size']

y = [33,40,43,44,48,51,100]

plt.xlim([0,105])

plt.title("Feature importance of KNN", fontsize=15, fontname="Times New Roman")

plt.barh(labels, width=y, height=0.8,color='pink')

plt.xticks(fontsize=12)

plt.yticks(fontsize=12)

plt.savefig('KNN.tiff',dpi=300)

fig = plt.gcf()

fig.set_size_inches(8,8)

labels = ['Grade','N-stage','Tumor Deposits','PI','T-stage','CEA','Tumor.size']

y = [5,10,15,20,25,51,100]

plt.xlim([0,105])

plt.title("Feature importance of SVM", fontsize=15, fontname="Times New Roman")

plt.barh(labels, width=y, height=0.8,color='green')

plt.xticks(fontsize=12)

plt.yticks(fontsize=12)

plt.savefig('SVM.tiff',dpi=300)

###################################################################

###################################################################

###################################################################

###################################################################

###################################################################

##

from sklearn.calibration import calibration_curve

def calibration_curve_1(k,y_pred,y_true,method_name,color,title):

prob_true, prob_pred = calibration_curve(y_true, y_pred, n_bins=5)

plt.figure(k)

plt.plot(prob_pred,prob_true,color=color,label='%s calibration_curve'%method_name,marker='s')

plt.plot([i/100 for i in range(0,100)],[i/100 for i in range(0,100)],color='black',linestyle='--')

plt.xlim(0,1.1,0.2)

plt.ylim(0,1.1,0.2)

plt.xlabel('y_preds')

plt.ylabel('y_real')

plt.title(title)

plt.legend(loc='lower right')

fig = plt.gcf()

fig.set_size_inches(8,8)

# plt.subplot(1,3,1)

# calibration_curve_1(1,modelLR.predict_proba(Xtrain[f_top])[:,1],Ytrain,'LR','red','train calibration_curve')

# calibration_curve_1(1,modelBNB.predict_proba(Xtrain[f_top])[:,1],Ytrain,'BNB','blue','train calibration_curve')

# calibration_curve_1(1,modelRF.predict_proba(Xtrain[f_top])[:,1],Ytrain,'RF','m','train calibration_curve')

# calibration_curve_1(1,modelDT.predict_proba(Xtrain[f_top])[:,1],Ytrain,'DT','green','train calibration_curve')

# calibration_curve_1(1,modelMLP.predict_proba(Xtrain[f_top])[:,1],Ytrain,'MLP','tomato','train calibration_curve')

# calibration_curve_1(1,modelKNN.predict_proba(Xtrain[f_top])[:,1],Ytrain,'KNN','darkblue','train calibration_curve')

# calibration_curve_1(1,modelSVM.predict_proba(Xtrain[f_top])[:,1],Ytrain,'SVM','deepskyblue','train calibration_curve')

calibration_curve_1(1,modelXGB.predict_proba(Xtrain[f_top])[:,1],Ytrain,'XGB','deepskyblue','train calibration_curve')

plt.savefig('TrainCalibrate.tiff',dpi=1200)

##

fig = plt.gcf()

fig.set_size_inches(8,8)

# plt.subplot(1,3,2)

# calibration_curve_1(1,modelLR.predict_proba(Xtest[f_top])[:,1],Ytest,'LR','red','test calibration_curve')

# calibration_curve_1(1,modelBNB.predict_proba(Xtest[f_top])[:,1],Ytest,'BNB','blue','test calibration_curve')

# calibration_curve_1(1,modelRF.predict_proba(Xtest[f_top])[:,1],Ytest,'RF','m','test calibration_curve')

calibration_curve_1(1,modelXGB.predict_proba(Xtest[f_top])[:,1],Ytest,'XGB','green','test calibration_curve')

# calibration_curve_1(1,modelMLP.predict_proba(Xtest[f_top])[:,1],Ytest,'MLP','tomato','test calibration_curve')

# calibration_curve_1(1,modelKNN.predict_proba(Xtest[f_top])[:,1],Ytest,'KNN','darkblue','test calibration_curve')

# calibration_curve_1(1,modelSVM.predict_proba(Xtest[f_top])[:,1],Ytest,'SVM','deepskyblue','test calibration_curve')

# calibration_curve_1(1,modelDT.predict_proba(Xtest[f_top])[:,1],Ytest,'DT','deepskyblue','test calibration_curve')

plt.savefig('TestCalibrate.tiff',dpi=1200)

##

fig = plt.gcf()

fig.set_size_inches(8,8)

# plt.subplot(1,3,3)

# calibration_curve_1(1,modelLR.predict_proba(Xtest[f_top])[:,1],Ytest,'LR','red','valid calibration_curve')

# calibration_curve_1(1,modelBNB.predict_proba(Xtest[f_top])[:,1],Ytest,'BNB','blue','valid calibration_curve')

# calibration_curve_1(1,modelRF.predict_proba(Xtest[f_top])[:,1],Ytest,'RF','m','valid calibration_curve')

# calibration_curve_1(1,modelMLP.predict_proba(Xtest[f_top])[:,1],Ytest,'MLP','tomato','valid calibration_curve')

# calibration_curve_1(1,modelKNN.predict_proba(Xtest[f_top])[:,1],Ytest,'KNN','darkblue','valid calibration_curve')

# calibration_curve_1(1,modelSVM.predict_proba(Xtest[f_top])[:,1],Ytest,'SVM','deepskyblue','valid calibration_curve')

# calibration_curve_1(1,modelDT.predict_proba(Xtest[f_top])[:,1],Ytest,'DT','deepskyblue','valid calibration_curve')

calibration_curve_1(1,modelXGB.predict_proba(Xvalid[f_top])[:,1],Yvalid,'XGB','tomato','valid calibration_curve')

plt.savefig('ValidCalibrate.tiff',dpi=1200)

###################################################################

###################################################################

###################################################################

###################################################################

###################################################################

##

col=['XGB','RF','DT','MLP','KNN','SVM','KNN','LR','Actual']

color=['#FFFACD','#E8222D']

a=0.1

# a=Optimal_threshold(-train['OSTA_score'],train['osteoporosis'])[1]

# pre_label2=[int(-i>a) for i in train['OSTA_score'].tolist()]

tmp1=pd.DataFrame([modelXGB.predict(Xtrain[f_top]),

modelRF.predict(Xtrain[f_top]),

modelDT.predict(Xtrain[f_top]),

modelMLP.predict(Xtrain[f_top]),

modelKNN.predict(Xtrain[f_top]),

modelSVM.predict(Xtrain[f_top]),

modelKNN.predict(Xtrain[f_top]),

modelLR.predict(Xtrain[f_top]),

# DecisionTree.predict(train[f_top]),

# rf.predict(train[f_top]),

# lr.predict(train[f_top]),

# mlp.predict(train[f_top]),

# BNB.predict(train[f_top]),

# pre_label2,

Ytrain],index=col).T

fig = plt.gcf() # gcf: get current figure调整图形大小

fig.set_size_inches(20,12)

plt.subplot(1,3,1)

sns.heatmap(data=tmp1,

vmax=1,

vmin=0,cmap = color,yticklabels=False,cbar=False

# cmap='YlOrRd_r',

# annot=True,

# fmt=".3f",

)

# sns.cubehelix_palette(as_cmap=True, reverse=True)

plt.title('Training set')

# plt.ylabel('N=%s'%Xtrain.shape[0],fontsize=20, color='k') #y轴label的文本和字体大小

plt.ylabel("N=19026",fontsize=20, color='k') #y轴label的文本和字体大小

plt.subplot(1,3,2)

col=['XGB','RF','DT','MLP','KNN','SVM','KNN','LR','Actual']

color=['#FFFACD','#E8222D']

a=0.1

# a=Optimal_threshold(-train['OSTA_score'],train['osteoporosis'])[1]

# pre_label2=[int(-i>a) for i in train['OSTA_score'].tolist()]

tmp2=pd.DataFrame([modelXGB.predict(Xtest[f_top]),

modelRF.predict(Xtest[f_top]),

modelDT.predict(Xtest[f_top]),

modelMLP.predict(Xtest[f_top]),

modelKNN.predict(Xtest[f_top]),

modelSVM.predict(Xtest[f_top]),

modelKNN.predict(Xtest[f_top]),

modelLR.predict(Xtest[f_top]),

# DecisionTree.predict(train[f_top]),

# rf.predict(train[f_top]),

# lr.predict(train[f_top]),

# mlp.predict(train[f_top]),

# BNB.predict(train[f_top]),

# pre_label2,

Ytest],index=col).T

fig = plt.gcf() # gcf: get current figure调整图形大小

fig.set_size_inches(12,12)

sns.heatmap(data=tmp2,

vmax=1,

vmin=0,cmap = color,yticklabels=False,cbar=False

# cmap='YlOrRd_r',

# annot=True,

# fmt=".3f",

)

# sns.cubehelix_palette(as_cmap=True, reverse=True)

plt.title('Testing set')

# plt.ylabel('N=%s'%Xtrain.shape[0],fontsize=20, color='k') #y轴label的文本和字体大小

plt.ylabel("N=8154",fontsize=20, color='k') #y轴label的文本和字体大小

##

plt.subplot(1,3,3)

col=['XGB','RF','DT','MLP','KNN','SVM','KNN','LR','Actual']

color=['#FFFACD','#E8222D']

a=0.1

tmp3=pd.DataFrame([modelXGB.predict(Xvalid[f_top]),

modelRF.predict(Xvalid[f_top]),

modelDT.predict(Xvalid[f_top]),

modelMLP.predict(Xvalid[f_top]),

modelKNN.predict(Xvalid[f_top]),

modelSVM.predict(Xvalid[f_top]),

modelKNN.predict(Xvalid[f_top]),

modelLR.predict(Xvalid[f_top]),

# DecisionTree.predict(train[f_top]),

# rf.predict(train[f_top]),

# lr.predict(train[f_top]),

# mlp.predict(train[f_top]),

# BNB.predict(train[f_top]),

# pre_label2,

Yvalid],index=col).T

fig = plt.gcf() # gcf: get current figure调整图形大小

fig.set_size_inches(20,12)

sns.heatmap(data=tmp3,

vmax=1,

vmin=0,cmap = color,yticklabels=False,cbar=False

# cmap='YlOrRd_r',

# annot=True,

# fmt=".3f",

)

# sns.cubehelix_palette(as_cmap=True, reverse=True)

plt.title('Valid set')

# plt.ylabel('N=%s'%Xtrain.shape[0],fontsize=20, color='k') #y轴label的文本和字体大小

plt.ylabel("N=1118",fontsize=20, color='k') #y轴label的文本和字体大小

plt.savefig('ActualHeatmap.tiff',dpi=1200)

###################################################################

###################################################################

###################################################################

###################################################################

###################################################################

##

def bootstrap_auc(y, pred, classes, bootstraps = 1000, fold_size = 10000):

statistics = np.zeros((len(classes), bootstraps))

for c in range(len(classes)):

df = pd.DataFrame(columns=['y', 'pred'])

# df.

df.loc[:, 'y'] = y

df.loc[:, 'pred'] = pred

df_pos = df[df.y == 1]

df_neg = df[df.y == 0]

prevalence = len(df_pos) / len(df)

for i in range(bootstraps):

pos_sample = df_pos.sample(n = int(fold_size * prevalence), replace=True)

neg_sample = df_neg.sample(n = int(fold_size * (1-prevalence)), replace=True)

y_sample = np.concatenate([pos_sample.y.values, neg_sample.y.values])

pred_sample = np.concatenate([pos_sample.pred.values, neg_sample.pred.values])

score = metrics.roc_auc_score(y_sample, pred_sample)

statistics[c][i] = score

return statistics

# print("均值:",np.mean(statistics,axis=1))

# print("最大值:",np.max(statistics,axis=1))

# print("最小值:",np.min(statistics,axis=1))

# train -训练

for model,name in zip([modelXGB,modelBNB,modelRF,modelMLP,modelKNN,modelLR,modelDT,modelSVM],['XGB','BNB','RF','MLP','KNN','LR','DT',"SVM"]):

statistics = bootstrap_auc(Yvalid,model.predict_proba(Xvalid)[:,1],[0,1])

print(name+"-OR (95% CI):",round(np.mean(statistics,axis=1)[1],4),'(',round(np.min(statistics,axis=1)[1],4),'-',round(np.max(statistics,axis=1)[1],4))

#####

##

from sklearn.metrics import brier_score_loss as BS

for model,name in zip([modelXGB,modelBNB,modelRF,modelMLP,modelKNN,modelLR,modelDT,modelSVM],['XGB','BNB','RF','MLP','KNN','LR','DT',"SVM"]):

print(name,'brier_score',BS(Ytrain,model.predict_proba(Xtrain)[:,1],pos_label = 1))

##

import pickle

with open('model.txt2','wb') as fq:

pickle.dump(modelXGB,fq)
